# Late-Onset Preeclampsia Is Linked to Extensive Remodeling of the Placental Extracellular Matrix

**DOI:** 10.3390/medsci14030364

**Published:** 2026-07-01

**Authors:** Cielo García-Montero, Tatiana Pekarek, Óscar Fraile-Martinez, Diego Liviu Boaru, Patricia de Castro-Martinez, Beatriz García-González, Marina Fanega-Fernández, Coral Bravo, Juan A. De Leon-Luis, Raul Diaz-Pedrero, Laura Lopez-Gonzalez, Moises Fernandez-Ibañez, Carlota Castilla, Silvestra Barrena-Blázquez, Julia Bujan, Natalio García-Honduvilla, Melchor Alvarez-Mon, Miguel A. Saez, Miguel A. Ortega

**Affiliations:** 1 Department of Medicine and Medical Specialities, Faculty of Medicine and Health Sciences, University of Alcalá, 28801 Alcalá de Henares, Spain; cielo.gmontero@gmail.com (C.G.-M.); tatianapekarek@gmail.com (T.P.); oscarfra.7@gmail.com (Ó.F.-M.); diegoboaru@hotmail.com (D.L.B.); patriciadecastro1999@gmail.com (P.d.C.-M.); beatriz.garciagonzal@edu.uah.es (B.G.-G.); marina.fanega@edu.uah.es (M.F.-F.); moisesfer1998@gmail.com (M.F.-I.); carlota.castilla@edu.uah.es (C.C.); mjulia.bujan@uah.es (J.B.); natalio.garcia@uah.es (N.G.-H.); mademons@gmail.com (M.A.-M.); msaega1@oc.mde.es (M.A.S.); 2 Ramón y Cajal Institute of Sanitary Research (IRYCIS), 28034 Madrid, Spain; raul.diazp@uah.es (R.D.-P.); laura.lgonzalez@uah.es (L.L.-G.); silvestra.barrena@uah.es (S.B.-B.); 3 Department of Public and Maternal and Child Health, School of Medicine, Complutense University of Madrid, 28040 Madrid, Spain; cbravoarribas@gmail.com (C.B.); jaleon@ucm.es (J.A.D.L.-L.); 4 Department of Obstetrics and Gynecology, University Hospital Gregorio Marañón, 28007 Madrid, Spain; 5 Health Research Institute Gregorio Marañón, 28009 Madrid, Spain; 6 Department of Surgery, Medical and Social Sciences, Faculty of Medicine and Health Sciences, University of Alcalá, 28801 Alcalá de Henares, Spain; 7 Department of Nursing and Physiotherapy, Faculty of Medicine and Health Sciences, University of Alcalá, 28801 Alcalá de Henares, Spain; 8 Immune System Diseases-Rheumatology and Internal Medicine Service, University Hospital Príncipe de Asturias, 28806 Alcalá de Henares, Spain; 9 Pathological Anatomy Service, Central University Hospital of Defence-UAH Madrid, 28801 Alcalá de Henares, Spain

**Keywords:** late-onset preeclampsia (LO-PE), histopathology, extracellular matrix (ECM), elastogenesis biomarkers, fibrillar collagens, matrix remodeling, lysyl oxidases, epidermal growth factor-like domain 7 (EGFL7), correlation analysis

## Abstract

Background: Late-onset preeclampsia (LO-PE) is the most prevalent clinical phenotype of preeclampsia and, although traditionally considered less strongly associated with placental dysfunction than early-onset disease, increasing evidence supports the presence of relevant placental alterations. The extracellular matrix (ECM) is a key regulator of villous architecture, tissue mechanics, trophoblast behavior, vascular remodeling, and angiogenesis. This study aimed to characterize ECM remodeling in placentas from women with LO-PE. Patients and Methods: A prospective observational study was conducted in 111 pregnant women, including 68 with LO-PE and 43 healthy controls. Placental samples were collected immediately after delivery. Gene expression of elastogenesis-related markers, cross-linking enzymes, fibrillar collagens, matrix-remodeling regulators, and endothelial–matrix signaling molecules was assessed by RT-qPCR. Protein expression was evaluated by immunohistochemistry. Differences between groups were analyzed using non-parametric tests with Benjamini–Hochberg correction, and correlations among ECM markers were explored using Spearman analysis. Results: LO-PE placentas showed significantly increased expression of tropoelastin (TE), fibulin-4 (FBLN-4), fibulin-5 (FBLN-5), fibrillin-1 (FBN-1), lysyl oxidase (LOX), lysyl oxidase-like 1 (LOXL-1), collagen type I (COL-I), collagen type III (COL-III), and matrix metalloproteinase-2 (MMP-2) at both gene and protein levels. Conversely, gene and protein expression of tissue inhibitor of metalloproteinase-2 (TIMP-2) and epidermal growth factor-like domain 7 (EGFL7) showed a marked decrease in the placentas of pregnant women with LO-PE. These findings indicate enhanced elastogenesis, increased matrix cross-linking, greater fibrillar collagen deposition, and an imbalance in matrix turnover. Correlation analysis further suggested that, although the FBLN-4/FBLN-5 axis remained preserved, LO-PE placentas displayed partial disruption of the broader ECM transcriptional network. Conclusions: LO-PE placentas exhibit a coordinated but dysregulated ECM remodeling phenotype involving elastic, collagenous, proteolytic, and endothelial–matrix regulatory pathways. These alterations support ECM remodeling as a relevant biological feature of LO-PE placental pathophysiology.

## 1. Introduction

Preeclampsia (PE) is a multisystem hypertensive disorder unique to human pregnancy, approximately affecting 2–8% of pregnancies and remaining as a major cause of maternal, fetal, and neonatal morbidity and mortality [1,2]. Clinically, PE is currently defined by new-onset hypertension after 20 weeks’ gestation accompanied by proteinuria and/or evidence of maternal organ dysfunction or uteroplacental dysfunction [3]. In addition, the clinical relevance of this entity extends beyond these changes, as PE is also associated with significant short- and long-term consequences for both mother and offspring, including increased cardiovascular risk later in life [4,5]. PE is commonly subclassified into early- and late-onset phenotypes using 34 weeks of gestation as the conventional threshold [6]. Early-onset preeclampsia (EO-PE) is typically associated with more severe clinical manifestations and a stronger link to impaired placentation, whereas late-onset preeclampsia (LO-PE) represents the predominant phenotype in clinical practice [7,8]. Despite its comparatively milder presentation, LO-PE is far from a benign condition and contributes substantially to the overall burden of disease due to its higher prevalence [9,10]. Notably, current predictive models and screening strategies show reduced understanding of LO-PE compared to EO-PE [11], underscoring important gaps in its early identification and mechanistic understanding.

The placenta plays a central role in the pathophysiology of PE, as placental stress and dysfunction are widely considered to both initiate and sustain the maternal syndrome through the release of anti-angiogenic, pro-inflammatory, oxidative, and trophoblast-derived mediators into maternal circulation [12]. In this context, recent integrative frameworks—particularly the concept of placental “age-mismatch”—have been proposed to explain the biological differences between EO-PE and LO-PE, suggesting that the timing between placental development and maternal adaptation is a key determinant of disease expression [13]. Although LO-PE has traditionally been viewed as a phenotype less tightly linked to primary placental insufficiency, accumulating evidence indicates that placental involvement remains substantial [14,15]. Indeed, LO-PE has been hypothesized to arise, at least in part, from maternal metabolic or cardiovascular burden leading to syncytiotrophoblast stress, and nearly half of LO-PE placentas exhibit lesions consistent with maternal vascular malperfusion, which have been associated with a more pronounced imbalance in angiogenic signaling [16]. In parallel, histopathological and molecular studies indicate that LO-PE placentas display a spectrum of tissue alterations compared to healthy pregnancies, including increased oxidative stress, ferroptosis, autophagy, microcalcifications, and inflammatory activation [17,18,19,20], supporting the presence of an active, multifactorial placental pathology. Collectively, these findings suggest that beyond classical vascular and angiogenic alterations, structural and biomechanical remodeling of placental tissue may represent an additional and underexplored dimension of LO-PE pathophysiology.

In this regard, the placental extracellular matrix (ECM) should not be viewed as a passive scaffold but rather as a dynamic regulator of villous architecture, tissue mechanics, trophoblast migration, vascular remodeling, and angiogenesis at the maternal–fetal interface [21,22,23]. From a structural and functional perspective, the placental ECM can be understood as an integrated system in which elastin- and collagen-based networks interact to determine tissue elasticity, stiffness, and mechanotransduction. Elastic fibers constitute a genuine structural component of human placental stem villi and of the perivascular contractile sheath [24,25], and preeclamptic placentas have been reported to exhibit altered elastic fiber content and thickening of villous vessel walls [26], suggesting that elastic remodeling accompanies placental dysfunction. Within this framework, tropoelastin (TE) is particularly informative as the soluble precursor of elastin, reflecting the synthetic entry point of elastogenesis [27]. Fibulin 4 (FBLN-4) and FBLN-5 act as critical organizers of elastic–fiber assembly [28,29]. Notably, FBLN-5 is expressed in placental trophoblast compartments and promotes primary extravillous trophoblast outgrowth, underscoring its relevance to placentation [30], whereas enhanced FBLN-4 expression in the placenta has been reported under pathological pregnancies [31]. Fibrillin 1 (FBN-1) provides the microfibrillar scaffold upon which elastin is deposited and is abundantly distributed within the villous stroma [32,33], contributing to the elastic properties of placental tissue.

Importantly, elastogenesis is tightly coupled to collagen organization through enzymatic cross-linking. Lysyl oxidase (LOX) and LOX-like 1 (LOXL-1) are critical enzymes that stabilize both elastin and collagen fibers by promoting covalent cross-links [34,35]. Both LOX and LOXL-1 are abundantly expressed in placental tissue and fetal membranes, and their dysregulation has been reported in pathological pregnancies [36]. In this context, collagen type I (COL-1) and type III (COL-3) represent the principal fibrillar collagens determining stromal architecture and mechanical stiffness in different tissues such as the human placenta [37,38]. Importantly, an altered proportion of both types of collagen has been reported in the placental tissue of women suffering from different obstetric complications [39,40,41].

At the same time, ECM homeostasis depends on a finely tuned balance between synthesis and degradation. Matrix metalloproteinase-2 (MMP2) and its inhibitor TIMP-2 capture the proteolytic arm of this system, regulating ECM turnover, trophoblast invasion, and angiogenesis, and their imbalance is a recurrent feature of PE [42,43]. Finally, EGFL7 is an ECM-associated angiogenic factor expressed by placental endothelial and trophoblast cells, which is reduced in PE placentas and may operate at the interface between endothelial homeostasis and matrix remodeling [44,45].

In this background, we hypothesized that LO-PE is characterized by a coordinated remodeling of the placental ECM compartment rather than by isolated alterations in individual matrix components. Specifically, we proposed that placental tissue from women with LO-PE would exhibit a distinct expression profile encompassing key elastogenic structural proteins (TE, FBLN-4, FBLN-5, and FBN-1), cross-linking enzymes (LOX and LOXL-1), fibrillar collagens (COL-1 and COL-3), matrix-remodeling regulators (MMP2 and TIMP-2), and endothelial/trophoblast–matrix signaling molecules (EGFL7), when compared with healthy pregnancies. Accordingly, the aim of the present study was to characterize and compare gene and protein expression patterns of these markers in the placental tissue of women with LO-PE and healthy controls by using real-time quantitative PCR (RT-qPCR) and immunohistochemistry (IHC).

## 2. Patients and Methods

### 2.1. Study Design

This investigation was conducted as a prospective, observational, analytical study nested within a clinical cohort. The study population comprised 111 pregnant women in the third trimester of gestation, recruited from the Hospital Central de la Defensa de Madrid, Spain, where their pregnancies were clinically monitored. Eligible participants were enrolled from 32 weeks of gestation onwards and were classified into two study groups according to their clinical status. The late-onset preeclampsia group included 68 women diagnosed with LO-PE, whereas the control group consisted of 43 healthy pregnant women (HC-PW) with no previous or current history of preeclampsia or other identified pregnancy-related disease.

LO-PE was diagnosed following the criteria established by the American College of Obstetricians and Gynecologists (ACOG) Clinical Practice Guidelines for Gestational Hypertension and Preeclampsia [46]. Late-onset disease was defined as preeclampsia diagnosed at or after 34 weeks of gestation. In patients with PE, disease severity was defined by the presence of one or more of the following clinical or analytical features: systolic blood pressure ≥ 160 mmHg and/or diastolic blood pressure ≥ 110 mmHg confirmed after 15 min; proteinuria ≥ 2 g in a 24 h urine collection or estimated by the urine protein/creatinine ratio; oliguria ≤ 500 mL/24 h or a urine output rate < 0.5 mL/kg/h for 2 h; renal impairment, defined as serum creatinine >1.1 mg/dL or a twofold increase in serum creatinine in the absence of pre-existing renal disease; neurological or visual symptoms, including persistent severe headache unresponsive to analgesic treatment, blurred vision, diplopia, or amaurosis; acute pulmonary edema or cyanosis; epigastric or right upper quadrant pain; hepatic dysfunction, defined by transaminase levels at least twice the upper normal limit; hematological abnormalities such as thrombocytopenia (<100,000/mm^3^), disseminated intravascular coagulation, or hemolysis; and evidence of placental involvement with fetal consequences, including intrauterine growth restriction, abnormal umbilical artery Doppler findings, or fetal death. For the purposes of the present study, serum creatinine values above 1.1 mg/dL were considered indicative of severe preeclampsia.

The clinical and demographic characteristics of the study participants are summarized in Table 1. The control group included 43 healthy pregnant women, while the LO-PE group included 68 affected patients. Maternal age, nulliparity, gestational age at delivery, cesarean section rate, and placental weight were recorded and compared between groups. Clinical information was obtained from obstetric records and reviewed before sample classification.

The study was performed in accordance with Good Clinical Practice recommendations and the ethical principles set out in the Declaration of Helsinki, revised in 2013, and the Oviedo Convention of 1997. The principles of autonomy, beneficence, and non-maleficence were respected throughout the study. Ethical approval was obtained from the Clinical Research Ethics Committee of the Hospital Universitario Central de la Defensa–UAH, reference LIB 12/2022, dated 30 September 2022. Written informed consent was obtained from all participants before inclusion in the study.

### 2.2. Sample Collection and Processing

Placental tissue samples were obtained immediately after delivery. In order to improve the representativeness of the sampling and include material from different cotyledonary regions, each placenta was divided into five fragments. Fragments were collected from macroscopically preserved areas of placental tissue, avoiding regions with evident infarction, calcification, hematoma, or mechanical disruption when technically possible. These fragments were placed in sterile tubes containing Minimum Essential Medium (MEM) supplemented with 1% antibiotic/antimycotic solution, both supplied by Thermo Fisher Scientific, Waltham, MA, USA. Samples were kept refrigerated and transported to the laboratory within two hours of delivery.

All subsequent procedures were carried out under sterile conditions in a class II laminar flow cabinet (Telstar AV 30/70 Müller 220 V 50 MHz, Telstar SA Group, Terrassa, Spain). Placental samples preserved in MEM were then processed for histopathological evaluation and immunodetection assays. When available, parallel tissue fragments were also used for molecular analyses.

For tissue preparation, placental fragments were further divided into smaller pieces and fixed in F13 solution, composed of 60% ethanol, 20% methanol, 7% polyethylene glycol, and 13% distilled water, in order to facilitate the removal of residual blood cells. After fixation, samples were embedded in paraffin using standard molds. Once the paraffin had solidified, serial sections of 5 µm thickness were obtained using an HM 350 S rotary microtome (Thermo Fisher Scientific, Waltham, MA, USA). Tissue sections were then floated on a warm water bath and mounted onto glass slides previously treated with 10% polylysine to enhance section adherence.

### 2.3. Protein Expression Analysis by Immunohistochemistry

Protein expression was assessed by immunohistochemistry following previously established protocols [17,41]. Antigen–antibody detection was performed using the avidin–biotin complex (ABC) method, with peroxidase as the enzymatic chromogenic system.

Primary antibodies, detailed in Table 2 and supplied by Abcam, Cambridge, UK, were diluted in phosphate-buffered saline (PBS) containing 3% bovine serum albumin (BSA). Sections were incubated with the corresponding primary antibody overnight at 4 °C. After this step, samples were incubated with a biotinylated secondary antibody diluted in PBS for 90 min at room temperature.

Immunoreactivity was visualized using diaminobenzidine as a chromogenic substrate, DAB Kit SK-4100, Vector Laboratories, Burlingame, CA, USA. The DAB working solution was freshly prepared immediately before use by mixing 5 mL of distilled water with two drops of buffer, four drops of DAB, and two drops of hydrogen peroxide. Sections were incubated with this solution for 60 min at room temperature, using a 1:200 dilution in PBS. Positive immunostaining was identified by the development of a brown precipitate.

For each immunohistochemical assay, negative controls were processed using sections from the same tissue samples. In these controls, incubation with the primary antibody was replaced by incubation with PBS/blocking solution. Immunostaining evaluation was independently performed by two histologists, M.A.O. and M.A.S., who were blinded to the study outcome. To maintain blinding, tissue sections were coded before microscopic evaluation, and the evaluators did not have access to the clinical group assignment during image assessment and data recording. Group allocation was only disclosed after completion of the immunohistochemical evaluation. Histological sections were examined using a Carl Zeiss Axiophot optical microscope (Jena, Germany). Representative areas of placental villous tissue were evaluated, with particular attention to the localization and intensity of immunostaining in stromal and vascular compartments. For each marker, immunohistochemical expression was quantified as the percentage of positively stained placental villi among the villi evaluated in each sample.

### 2.4. Gene Expression Analysis by Real-Time Quantitative PCR

Target gene expressions were evaluated by quantitative reverse transcription polymerase chain reaction (RT-qPCR). Total RNA was extracted using the guanidine–phenol–chloroform isothiocyanate method [47]. After RNA extraction, complementary DNA (cDNA) was obtained and quantified using Thermo Fisher Scientific reagents/equipment. Specific primers were designed using NCBI Primer BLAST, incorporating Primer3 version 2.5.0, and AutoDimer version 1.0 [48,49] to improve target specificity and reduce the probability of primer dimer formation.

Quantitative PCR was performed using a StepOnePlus™ system and the relative standard curve method. For each reaction, samples were first diluted in nuclease-free water. A final reaction volume of 20 µL was prepared by combining 5 µL of diluted sample, 10 µL of iQ™ SYBR^®^ Green Supermix intercalating reagent (Bio-Rad Laboratories, Hercules, CA, USA), 1 µL of forward primer, 1 µL of reverse primer, and 3 µL of DNase- and RNase-free water. Reactions were loaded onto MicroAmp^®^ 96-well plates (Applied Biosystems-Life Technologies, Foster City, CA, USA).

Glyceraldehyde 3-phosphate dehydrogenase (GAPDH) and TATA-box binding protein (TBP) were used as the housekeeping genes for normalization of gene expression data (Table 3). The values obtained for each target gene were interpolated from the corresponding standard curve. Standard curves were run in duplicate, while all samples were analyzed in triplicate. Negative controls were included in the remaining wells to monitor potential contamination or non-specific amplification.

### 2.5. Statistical Analysis

Statistical analyses were performed using R software (version 2026.01.0 + 392). Data distribution and homoscedasticity were assessed to determine the use of non-parametric tests. Descriptive statistics are presented as median and interquartile range (IQR).

Differences in gene expression levels between the HC and LO-PE groups were evaluated using the Wilcoxon rank-sum test (also known as the Mann–Whitney U test), as implemented in the stats package. To control Type I errors arising from multiple testing across the eleven markers, *p*-values were adjusted using the Benjamini–Hochberg False Discovery Rate (FDR) method.

Relationships between the expression levels of the extracellular matrix (ECM) markers (TE, FBLN_4, FBLN_5, FBN_1, LOX, LOXL_1, COL_I, COL_III, MMP2, TIMP2, and EGFL7) were assessed using Spearman’s rank correlation coefficient (ρ). This non-parametric approach was chosen to capture monotonic relationships regardless of the data distribution. Correlation matrices and *p*-values were calculated using the *Hmisc* package.

Data manipulation and tidying were conducted using the *tidyverse* suite, specifically *dplyr* and *tidyr*. Correlograms were generated using the *corrplot* package to visualize the strength and direction of the associations. In these plots: (1) the color intensity and numerical values represent the Spearman correlation coefficient; (2) the order of the markers was kept consistent across groups to facilitate direct comparison; (3) statistical significance was indicated by asterisks: * *p* < 0.05, *p* < 0.01, and *** *p* < 0.001 after FDR correction.

All tests were two-tailed, and an adjusted *p* < 0.05 was considered statistically significant.

## 3. Results

### 3.1. The Placentas of Women with LO-PE Exhibit Increased Expression of TE, FBLN-4, FBLN-5 and FBN-1

First, we examined the gene and protein expression of elastin-related biomarkers. The values obtained from the RT-qPCR are summarized in Supplementary Material Table S1, and the immunohistochemical expression data appears in Supplementary Material Table S2.

**Figure 1:**
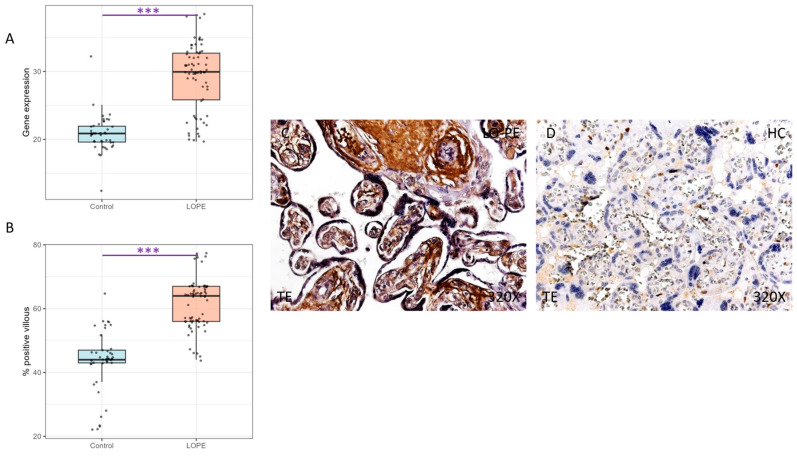
Tropoelastin (TE) expression in placental villi from pregnant women with late-onset preeclampsia (LO-PE) and healthy controls (HCs). (**A**) TE mRNA expression in LO-PE and HC placentas. (**B**) Percentage of TE-positive placental villi in the LO-PE and HC groups. (**C**,**D**) Representative images showing immunostaining for TE in placental villi from LO-PE and HC samples. *p* < 0.001 (***).

RT-qPCR analysis showed a significant increase in TE gene expression in placental tissue from women with LO-PE compared with healthy controls (LO-PE = 29.938 [25.824–32.686]; HC = 20.867 [19.606–21.939], *** adjusted *p* < 0.0001, Figure 1A). In line with these transcriptional findings, immunohistochemical assessment revealed a significantly higher percentage of TE-positive placental villi in the LO-PE group (LO-PE = 64 [56–67]; HC = 44 [43–47], *** adjusted *p* < 0.0001, Figure 1B). Representative microphotographs confirmed TE immunostaining within the placental villous compartment (Figure 1C,D).

**Figure 2:**
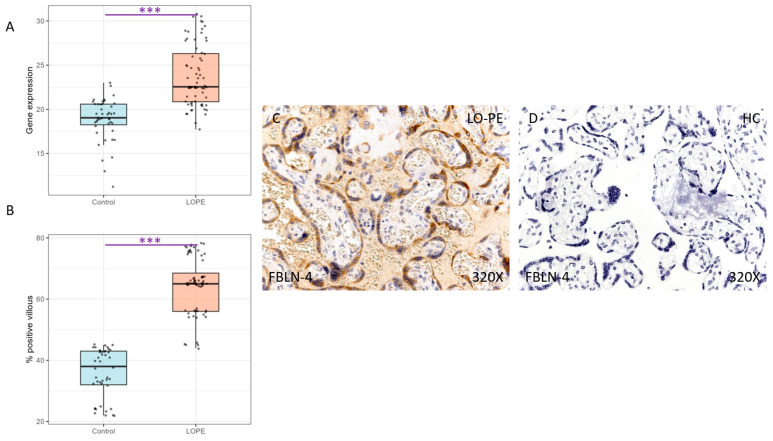
Fibulin 4 (FBLN-4) expression in placental villi from pregnant women with late-onset preeclampsia (LO-PE) and healthy controls (HCs). (**A**) FBLN-4 mRNA expression in LO-PE and HC placentas. (**B**) Percentage of FBLN-4-positive placental villi in the LO-PE and HC groups. (**C**,**D**) Representative images showing immunostaining for FBLN-4 in placental villi from LO-PE and HC samples. *p* < 0.001 (***).

FBLN-4 gene expression was also significantly increased in LO-PE placentas compared with HC (LO-PE = 22.546 [20.855–26.304]; HC = 19.039 [18.245–20.584], *** adjusted *p* < 0.0001, Figure 2A). Consistently, IHC analysis showed a marked increase in the percentage of FBLN-4-positive villi in LO-PE samples (LO-PE = 65 [56–68.5]; HC = 38 [32–43], *** adjusted *p* < 0.0001, Figure 2B). Representative images illustrated stronger FBLN-4 immunoreactivity in placental villi from LO-PE cases compared with controls (Figure 2C,D).

**Figure 3:**
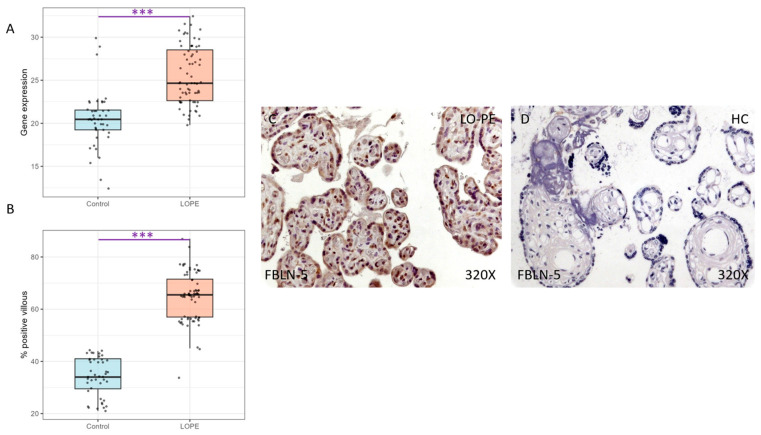
Fibulin 5 (FBLN-5) expression in placental villi from pregnant women with late-onset preeclampsia (LO-PE) and healthy controls (HCs). (**A**) FBLN-5 mRNA expression in LO-PE and HC placentas. (**B**) Percentage of FBLN-5-positive placental villi in the LO-PE and HC groups. (**C**,**D**) Representative images showing immunostaining for FBLN-5 in placental villi from LO-PE and HC samples. *p* < 0.001 (***).

Similarly, FBLN-5 expression was significantly upregulated at the mRNA level in placentas from women with LO-PE (LO-PE = 24.655 [22.634–28.524]; HC = 20.459 [19.238–21.540], *** adjusted *p* < 0.0001, Figure 3A). This increase was confirmed by immunohistochemistry, which demonstrated a higher percentage of FBLN-5-positive placental villi in LO-PE samples (LO-PE = 65.5 [57–71.5]; HC = 34 [29.5–41], *** adjusted *p* < 0.0001, Figure 3B). Representative tissue sections supported this augmented FBLN-5 expression pattern in LO-PE villi (Figure 3C,D).

**Figure 4:**
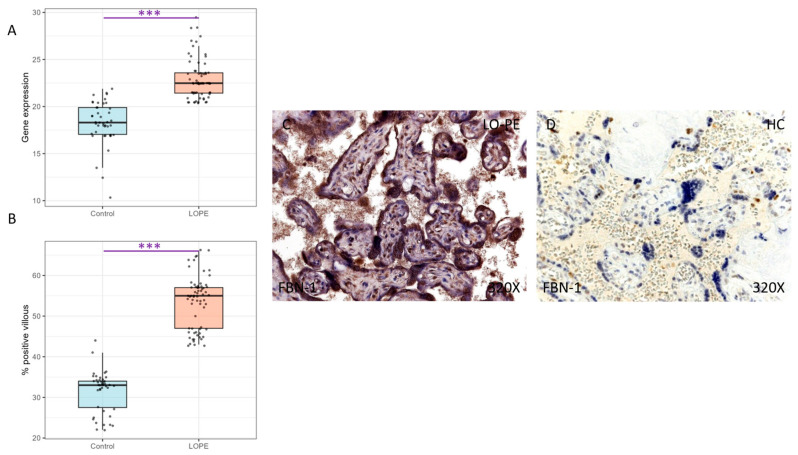
Fibrillin 1 (FBN-1) expression in placental villi from pregnant women with late-onset preeclampsia (LO-PE) and healthy controls (HCs). (**A**) FBN-1 mRNA expression in LO-PE and HC placentas. (**B**) Percentage of FBN-1-positive placental villi in the LO-PE and HC groups. (**C**,**D**) Representative images showing immunostaining for FBN-1 in placental villi from LO-PE and HC samples. *p* < 0.001 (***).

FBN-1 gene expression was significantly elevated in the placental tissue of women with LO-PE compared with HC (LO-PE = 22.483 [21.431–23.582]; HC = 18.299 [17.043–19.894], *** adjusted *p* < 0.0001, Figure 4A). In agreement, IHC quantification revealed a significant increase in the percentage of FBN-1-positive placental villi in the LO-PE group (LO-PE = 55 [47–57]; HC = 33 [27.5–34], *** adjusted *p* < 0.0001, Figure 4B). Representative images showed enhanced FBN-1 immunostaining in LO-PE placental villi compared with controls (Figure 4C,D).

### 3.2. The Placentas of Women with LO-PE Display Augmented Expression of LOX and LOXL-1

We next explored the gene and protein expression of LOX and LOXL-1. The values obtained from the RT-qPCR are summarized in Supplementary Material Table S1, and the immunohistochemical expression data appears in Supplementary Material Table S2.

**Figure 5:**
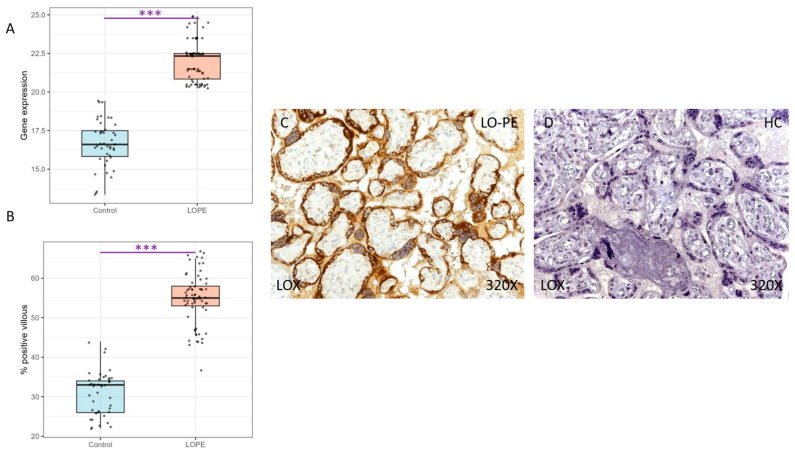
Lysyl oxidase (LOX) expression in placental villi from pregnant women with late-onset preeclampsia (LO-PE) and healthy controls (HCs). (**A**) LOX mRNA expression in LO-PE and HC placentas. (**B**) Percentage of LOX-positive placental villi in the LO-PE and HC groups. (**C**,**D**) Representative images showing immunostaining for LOX in placental villi from LO-PE and HC samples. *p* < 0.001 (***).

RT-qPCR analysis demonstrated a significant increase in LOX gene expression in placental tissue from women with LO-PE compared with HC (LO-PE = 22.329 [20.841–22.493]; HC = 16.599 [15.822–17.494], *** adjusted *p* < 0.0001, Figure 5A). Likewise, immunohistochemical quantification showed a significantly higher percentage of LOX-positive placental villi in LO-PE samples (LO-PE = 55 [53–58]; HC = 33 [26–34], *** adjusted *p* < 0.0001, Figure 5B). Representative images confirmed increased LOX immunoreactivity in placental villi from LO-PE patients (Figure 5C,D).

**Figure 6:**
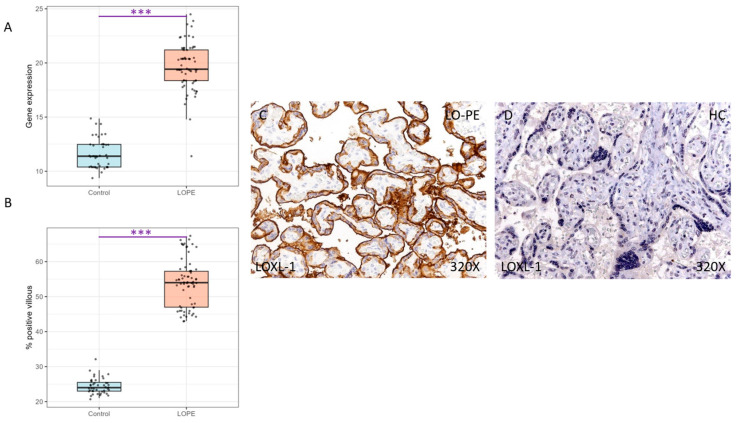
Lysyl oxidase-like 1 (LOXL1) expression in placental villi from pregnant women with late-onset preeclampsia (LO-PE) and healthy controls (HCs). (**A**) LOXL1 mRNA expression in LO-PE and HC placentas. (**B**) Percentage of LOXL1-positive placental villi in the LO-PE and HC groups. (**C**,**D**) Representative images showing immunostaining for LOXL1 in placental villi from LO-PE and HC samples. *p* < 0.001 (***).

LOXL-1 expression followed a similar pattern, showing marked upregulation in LO-PE placentas at the gene level (LO-PE = 19.423 [18.368–21.202]; HC = 11.389 [10.390–12.479], *** adjusted *p* < 0.0001, Figure 6A). Consistently, IHC analysis revealed a significantly greater percentage of LOXL-1-positive villi in LO-PE placentas compared with controls (LO-PE = 54 [47–57.25]; HC = 24 [23–25.5], *** adjusted *p* < 0.0001, Figure 6B). Representative microphotographs illustrated stronger LOXL-1 staining in LO-PE placental villi (Figure 6C,D).

### 3.3. The Placentas of Women with LO-PE Present Higher Expression of COL-1 and -3

Subsequently, the gene and protein expression of COL-1 and COL-3 was assessed. The values obtained from RT-qPCR are summarized in Supplementary Material Table S1, and the immunohistochemical expression data appears in Supplementary Material Table S2.

**Figure 7:**
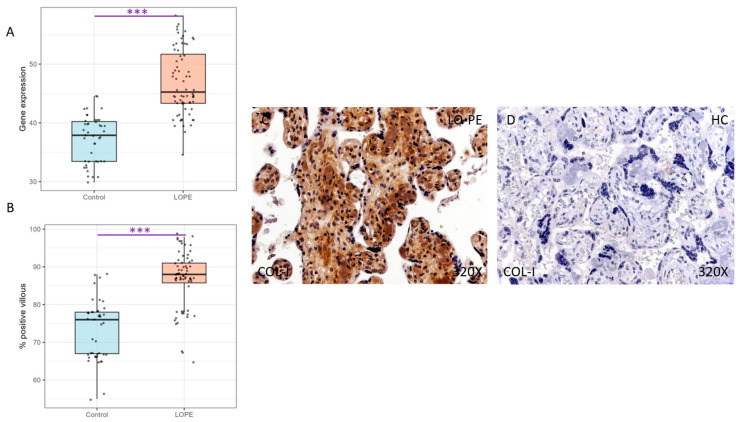
Collagen type I (COL-1) expression in placental villi from pregnant women with late-onset preeclampsia (LO-PE) and healthy controls (HCs). (**A**) COL-1 mRNA expression in LO-PE and HC placentas. (**B**) Percentage of COL-1-positive placental villi in the LO-PE and HC groups. (**C**,**D**) Representative images showing immunostaining for COL-1 in placental villi from LO-PE and HC samples. *p* < 0.001 (***).

RT-qPCR analysis revealed significantly increased COL-I gene expression in LO-PE placentas compared with HC (LO-PE = 45.245 [43.326–51.672]; HC = 37.890 [33.446–40.226], *** adjusted *p* < 0.0001, Figure 7A). This result was paralleled by IHC analysis, which showed a higher percentage of COL-I-positive placental villi in LO-PE samples (LO-PE = 88 [85.75–91]; HC = 76 [67–78], *** adjusted *p* < 0.0001, Figure 7B). Representative histological sections demonstrated increased COL-I immunostaining in LO-PE placental villi (Figure 7C,D).

**Figure 8:**
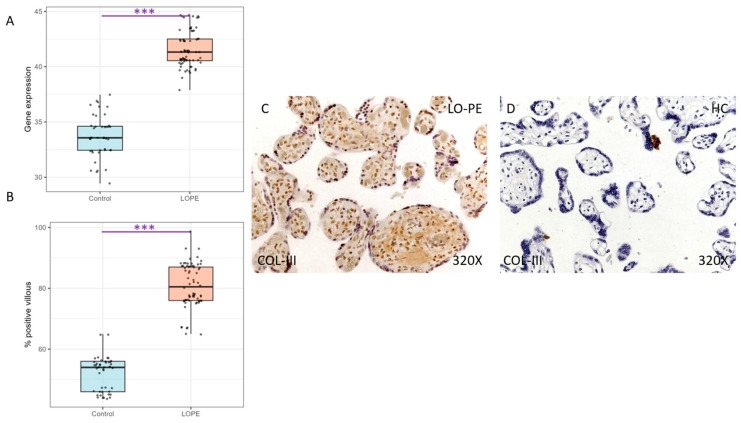
Collagen type III (COL-3) expression in placental villi from pregnant women with late-onset preeclampsia (LO-PE) and healthy controls (HCs). (**A**) COL-3 mRNA expression in LO-PE and HC placentas. (**B**) Percentage of COL-3-positive placental villi in the LO-PE and HC groups. (**C**,**D**) Representative images showing immunostaining for COL-3 in placental villi from LO-PE and HC samples. *p* < 0.001 (***).

COL-III gene expression was also significantly elevated in placental tissue from women with LO-PE (LO-PE = 41.324 [40.547–42.507]; HC = 33.566 [32.442–34.612], *** adjusted *p* < 0.0001, Figure 8A). In accordance with this finding, immunohistochemical quantification showed a marked increase in the percentage of COL-III-positive villi in the LO-PE group (LO-PE = 80.5 [76–87]; HC = 54 [46–56], *** adjusted *p* < 0.0001, Figure 8B). Representative images confirmed stronger COL-III immunoreactivity in placental villi from LO-PE cases compared with controls (Figure 8C,D).

### 3.4. The Placentas of Women with LO-PE Are Defined by a Marked Increase in MMP-2 with a Concomitant Decrease in Its Inhibitor TIMP-2

The expression of MMP-2 and its inhibitor TIMP-2 was equally studied in this work. The values obtained from the RT-qPCR are summarized in Supplementary Material Table S1, and the immunohistochemical expression data appears in Supplementary Material Table S2.

**Figure 9:**
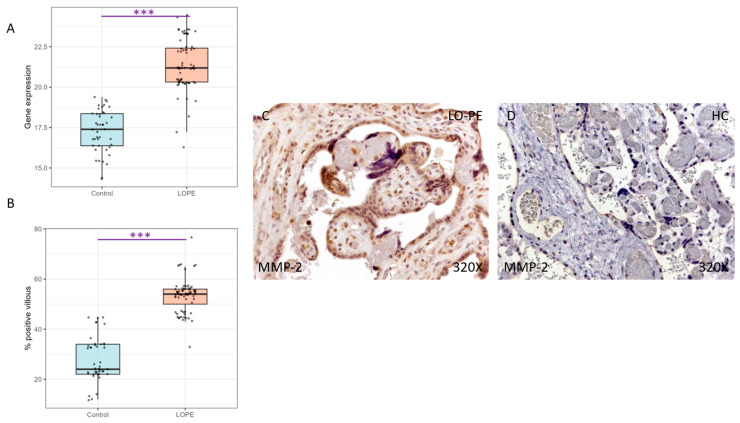
Matrix metalloproteinase 2 (MMP-2) expression in placental villi from pregnant women with late-onset preeclampsia (LO-PE) and healthy controls (HCs). (**A**) MMP-2 mRNA expression in LO-PE and HC placentas. (**B**) Percentage of MMP-2-positive placental villi in the LO-PE and HC groups. (**C**,**D**) Representative images showing immunostaining for MMP-2 in placental villi from LO-PE and HC samples. *p* < 0.001 (***).

RT-qPCR analysis showed that MMP-2 gene expression was significantly increased in LO-PE placentas compared with HC (LO-PE = 21.190 [20.318–22.415]; HC = 17.390 [16.373–18.358], *** adjusted *p* < 0.0001, Figure 9A). Immunohistochemical assessment confirmed this increase, revealing a significantly higher percentage of MMP-2-positive placental villi in LO-PE samples (LO-PE = 54 [50–56]; HC = 24 [22–34], *** adjusted *p* < 0.0001, Figure 9B). Representative microphotographs showed enhanced MMP-2 immunostaining in LO-PE placental villi (Figure 9C,D).

**Figure 10:**
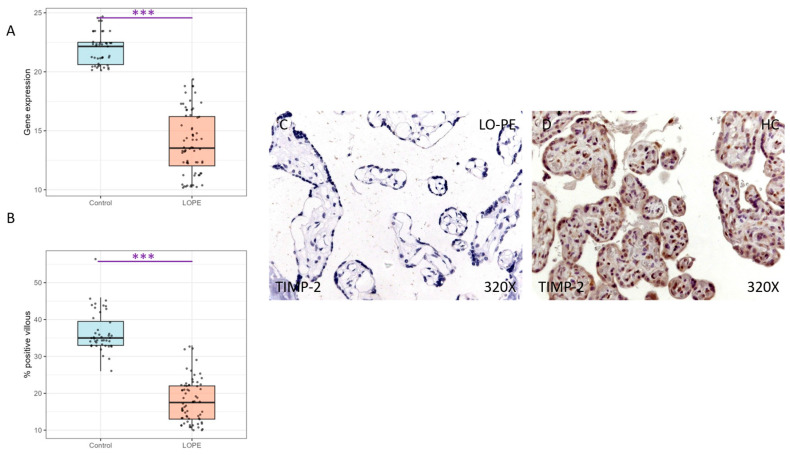
Tissue inhibitor of metalloproteinase 2 (TIMP-2) expression in placental villi from pregnant women with late-onset preeclampsia (LO-PE) and healthy controls (HCs). (**A**) TIMP-2 mRNA expression in LO-PE and HC placentas. (**B**) Percentage of TIMP-2-positive placental villi in the LO-PE and HC groups. (**C**,**D**) Representative images showing immunostaining for TIMP-2 in placental villi from LO-PE and HC samples. *p* < 0.001 (***).

In contrast, TIMP-2 gene expression was significantly reduced in placental tissue from women with LO-PE compared with HC (LO-PE = 13.529 [12.027–16.204]; HC = 22.149 [20.610–22.502], *** adjusted *p* < 0.0001, Figure 10A). This decrease was also observed at the protein level, as IHC analysis demonstrated a lower percentage of TIMP-2-positive placental villi in LO-PE samples (LO-PE = 17.5 [13–22]; HC = 35 [33–39.5], *** adjusted *p* < 0.0001, Figure 10B). Representative images further supported the reduced TIMP-2 immunoreactivity in the placental villous compartment of LO-PE cases (Figure 10C,D).

### 3.5. The Placentas of Women with LO-PE Are Characterized by a Noteworthy Reduction in EGFL-7

Finally, expression levels of EGFL-7 were detected in our study. The values obtained from the RT-qPCR are summarized in Supplementary Material Table S1, and the immunohistochemical expression data appears in Supplementary Material Table S2.

**Figure 11:**
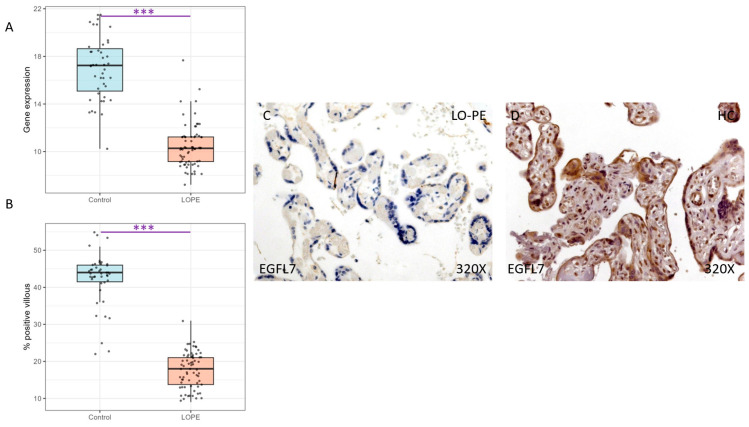
Epidermal growth factor-like domain 7 (EGFL-7) expression in placental villi from pregnant women with late-onset preeclampsia (LO-PE) and healthy controls (HCs). (**A**) EGFL-7 mRNA expression in LO-PE and HC placentas. (**B**) Percentage of EGFL-7-positive placental villi in the LO-PE and HC groups. (**C**,**D**) Representative images showing immunostaining for EGFL-7 in placental villi from LO-PE and HC samples. *p* < 0.001 (***).

RT-qPCR analysis demonstrated a significant decrease in EGFL-7 gene expression in placental tissue from women with LO-PE compared with HC (LO-PE = 10.279 [9.165–11.234]; HC = 17.239 [15.086–18.643], *** adjusted *p* < 0.0001, Figure 11A). In agreement with this transcriptional reduction, IHC quantification showed a markedly lower percentage of EGFL-7-positive placental villi in LO-PE samples (LO-PE = 18 [13.75–21]; HC = 44 [41.5–46], *** adjusted *p* < 0.0001, Figure 11B). Representative microphotographs confirmed reduced EGFL-7 immunostaining in LO-PE placental villi compared with controls (Figure 11C,D).

### 3.6. Co-Expression Patterns of ECM Markers

**Figure 12:**
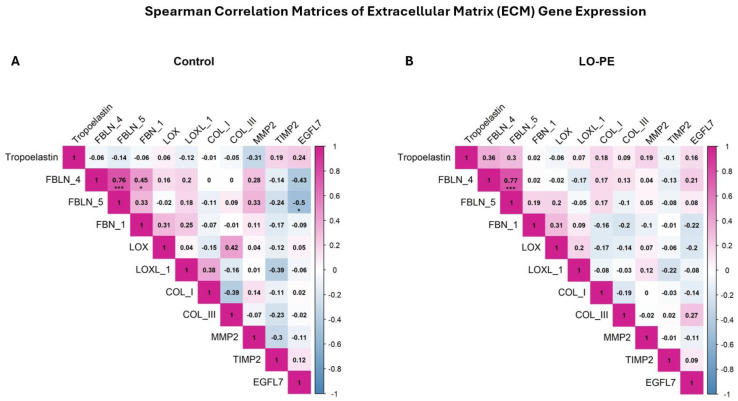
Spearman Correlation Matrices of Extracellular Matrix (ECM) Gene Expression. Correlograms representing the co-expression patterns of eleven ECM markers in the (**A**) Control group and (**B**) LO-PE group. The color scale indicates the Spearman correlation coefficient (ρ), ranging from −1 (blue, strong negative correlation) to +1 (magenta, strong positive correlation). Numerical values within each cell represent the ρ coefficient. Statistical significance, determined after Benjamini–Hochberg FDR correction, is indicated by asterisks: * *p* < 0.05 and *** *p* < 0.001. Non-significant correlations are shown without symbols.

Correlation analysis revealed a specific signature of co-regulation among the studied markers, which differed between groups. In the Control group (Figure 12A), a highly significant and strong positive correlation was identified between *FBLN_4* and *FBLN_5* (ρ = 0.76, *p* < 0.001). Additionally, *FBLN_4* showed a moderate positive association with *FBN_1* (ρ = 0.45, *p* < 0.05), while *FBLN_5* exhibited a significant moderate negative correlation with *EGFL7* (ρ = −0.50, *p* < 0.05).

In the LO-PE group (Figure 12B), the strong positive association between *FBLN_4* and *FBLN_5* remained stable and significant (ρ = 0.77, *p* < 0.001). However, the significant relationships observed in the Control group involving *FBN_1* and *EGFL7* were lost in the LO-PE subjects. Despite some moderate coefficients appearing in the matrix (e.g., *Tropoelastin* and *FBLN_4*, ρ = 0.36), these did not reach statistical significance after multiple testing correction. Overall, the LO-PE group displayed a more fragmented correlation profile, suggesting a potential disruption in the coordinated expression of these elastogenesis-related genes compared to the Control group.

## 4. Discussion

The present study identifies a coordinated ECM remodeling phenotype in placentas from pregnant women with LO-PE characterized by increased expression of elastogenesis-related markers (tropoelastin, FBLN-4, FBLN-5, and FBN-1), cross-linking enzymes (LOX and LOXL-1), fibrillar collagens (COL-1 and COL-3), and MMP2, together with decreased TIMP-2 and EGFL7. From a mechanistic perspective, this profile supports the view of a placenta that is not merely “injured,” but actively remodeled, with concurrent activation of elastic–fiber assembly, matrix cross-linking, collagen deposition, and unbalanced proteolysis. This interpretation aligns with current models of LO-PE, in which placental pathology may appear less overt than in early-onset disease, yet still reflects biologically meaningful dysregulation of various biological pathways [16,19,20,50,51,52,53]. Indeed, although term preeclampsia may show only limited conventional histopathological alterations in some cases, a substantial proportion of LO-PE placentas exhibit lesions consistent with maternal vascular malperfusion, and recent single-cell and proteomic studies increasingly implicate ECM remodeling, trophoblast dysfunction, and angiogenic imbalance in its pathobiology [54,55].

The observed upregulation of TE, FBLN-4, FBLN-5, and FBN-1 in LO-PE placentas aligns with evidence that elastogenic proteins are critical for maintaining placental elasticity and vascular integrity [56,57]. TE serves as the precursor for mature elastin fibers, while FBLN-4 and FBLN-5 act as essential chaperones facilitating elastic fiber assembly and cross-linking [31,58,59]. FBN-1 provides the microfibrillar scaffold necessary for elastin deposition [58]. Increased expression of these components has been reported not only in preeclampsia but also in conditions characterized by altered hemodynamics or vascular stress during pregnancy [31,60]. The coordinated upregulation may represent a compensatory response to increased mechanical or oxidative stress within the placenta, as reported in previous studies [18,61]. However, excessive or disorganized elastogenesis could disrupt normal tissue architecture and contribute to impaired placental perfusion [21].

LOX and its isoforms catalyze the cross-linking of collagen and elastin fibers, thereby modulating ECM stiffness. As such, increased LOX-family activity is expected to promote structural consolidation of the extracellular matrix once deposited. However, the role of LOX and its isoforms in trophoblast biology appears to be temporally and contextually regulated. Experimental evidence indicates that inhibition of LOX activity enhances trophoblast invasiveness under hypoxic conditions [62], suggesting that LOX may restrain invasion during early placentation. Conversely, reduced LOX signaling has been associated with impaired trophoblast invasion and abnormal collagen deposition in PE, particularly in EO-PE [36]. In LO-PE, we observed increased LOX and LOXL-1 expression in the placentas at delivery, suggesting a possible pathophysiological difference between these variants. Indeed, our results are consistent with studies indicating their upregulation in pathological pregnancies marked by abnormal vascular remodeling [31,35,36,63]. While increased LOX and LOXL activity may enhance ECM stability under physiological conditions, excessive cross-linking can lead to heightened tissue rigidity—a feature implicated in preeclampsia pathogenesis [64].

Collagens I and III are major structural proteins within the placental stroma [38]. Their increased expression in LO-PE suggests enhanced fibrotic remodeling, an increasingly recognized hallmark of PE reflecting active stromal remodeling driven by fibroblast activation and ECM accumulation rather than passive degeneration [65]. Among fibrillar collagens, collagen I appears particularly relevant, as its increased deposition may actively contribute to disease progression by altering trophoblast behavior and placental architecture [66]. Accordingly, in vitro models have shown that collagen I induces preeclampsia-like symptoms by suppressing trophoblast proliferation and invasion through the inhibition of ERK and WNT/β-catenin signaling pathways [66]. Although collagen III has been less extensively studied, classical histological analyses consistently identify both collagen I and III in villous fibrosis [41,67]. Accordingly, the combined increase in COL-1 and COL-3 observed in our LO-PE placentas likely reflects broad interstitial and perivascular ECM remodeling rather than selective alteration of a single collagen subtype. Functionally, this pattern would be expected to increase stromal density, reduce villous compliance, and reinforce the stiffened matrix state suggested by the concomitant rise in elastogenic and cross-linking markers.

In parallel, the MMP2/TIMP2 profile indicates that LO-PE placentas are not only fibrotic but also characterized by active and insufficiently regulated matrix turnover. MMP2 is a key gelatinase involved in ECM degradation and trophoblast invasion, particularly during the first trimester of pregnancy, whereas TIMP2 acts as its principal endogenous inhibitor [68,69]. While earlier studies frequently associated PE with reduced MMP-2 and MMP-9 activity and increased TIMP expression, more recent evidence highlights substantial heterogeneity across disease stages and phenotypes [70]. In particular, increased placental and serum MMP-2 levels appear to be common for both severe EO-PE and LO-PE, according to several studies [71,72,73]. Less evidence has specifically addressed the role of TIMP-2 in PE; however, it seems that a concomitant downregulation of this component is associated with gestational trophoblastic diseases and hypertensive disorders of pregnancy [74]. Conversely, other studies have demonstrated that increased serum TIMP-2 detection could act as an important predictor of PE, particularly at the second and third trimester of pregnancy [75,76]. In our study, the combination of increased MMP2 and decreased TIMP2 observed the placentas of women with LO-PE is consistent with a high-turnover remodeling state rather than a static fibrotic endpoint. Reduced TIMP2 likely amplifies the biological impact of elevated MMP2, favoring a proteolytic environment in which matrix degradation proceeds in a relatively unrestrained manner, as reported under pathological pregnancies [77]. This dynamic imbalance suggests that collagen accumulation and matrix breakdown coexist, contributing to structural disorganization rather than stable fibrosis.

On the other hand, the concomitant decrease in EGFL7 adds an important angiogenic dimension to this profile. EGFL7, expressed by trophoblast and endothelial cells, promotes migration, invasion, and vascular homeostasis [78]. Previous studies have reported that the placental expression of EGFL7 is reduced in women with PE, whereas increased serum detection of this component is reported, possibly due to increased mobilization of placenta-produced EGFL7 [79,80]. Its reduction at both gene and protein levels in our LO-PE placentas may support a local defect in endothelial–trophoblast signaling. Additionally, reduced EGFL7 may further impair vascular development while indirectly promoting elastogenic protein accumulation [81]. When integrated with the MMP2-high/TIMP2-low pattern, decreased EGFL7 suggests a matrix environment characterized by dysregulated proteolysis and impaired angiogenic competence. Coupled with the simultaneous increase in elastogenic, cross-linking, and fibrillar collagen components, this profile is most consistent with maladaptive remodeling rather than restoration of normal placental architecture.

Beyond these individual expression changes, the correlation analysis provides an additional layer of interpretation by suggesting that LO-PE is also associated with altered coordination within the ECM transcriptional network. The most consistent finding was the strong positive correlation between FBLN-4 and FBLN-5, which was preserved in both control and LO-PE placentas. This persistent association may indicate that the core fibulin module involved in elastic fiber assembly remains transcriptionally coupled, even under pathological conditions. This interpretation is consistent with previous evidence showing that FBLN-4 and FBLN-5 are essential, non-redundant contributors to elastic fiber assembly and ECM organization, acting in close relationship with tropoelastin, LOX-dependent cross-linking, and the fibrillin-rich microfibrillar scaffold [82,83]. Therefore, preservation of the FBLN-4/FBLN-5 association may reflect maintenance of a basic elastogenic assembly module, even in the context of placental disease.

However, this preserved relationship contrasted with a broader loss of network connectivity in LO-PE placentas. In control samples, elastogenesis-related markers showed a more integrated correlation structure, including associations between fibulins, FBN-1 and EGFL7, consistent with a coordinated ECM regulatory program. By contrast, the attenuation or disappearance of these associations in LO-PE suggests a partial transcriptional decoupling of the ECM network. Thus, LO-PE may not involve a complete collapse of ECM regulation, but rather a selective remodeling of network architecture, in which a core elastogenic module remains coupled while its broader integration with microfibrillar organization, vascular signaling, and matrix turnover becomes weakened. This interpretation is aligned with network-medicine concepts, where disease phenotypes are increasingly understood as alterations in molecular connectivity and pathway organization rather than only as isolated changes in individual molecules [84,85].

Therefore, the pathological phenotype observed in LO-PE may not depend solely on the upregulation or downregulation of individual ECM components but also on the loss of coordinated regulation among them. In this regard, the preserved FBLN-4/FBLN-5 axis should not be interpreted as evidence of normal elastogenesis but rather as a residual or compensatory module within an otherwise dysregulated ECM network. This may help explain how increased expression of elastogenic, collagenous and matrix-remodeling markers can coexist with impaired endothelial–matrix signaling, particularly considering the role of EGFL7 in trophoblast/endothelial biology and fetoplacental vascular development. This network-level disruption may help explain how increased expression of elastogenic, collagenous and matrix-remodeling markers can coexist with impaired endothelial–matrix signaling and ultimately contribute to a disorganized, maladaptive villous ECM architecture.

**Figure 13:**
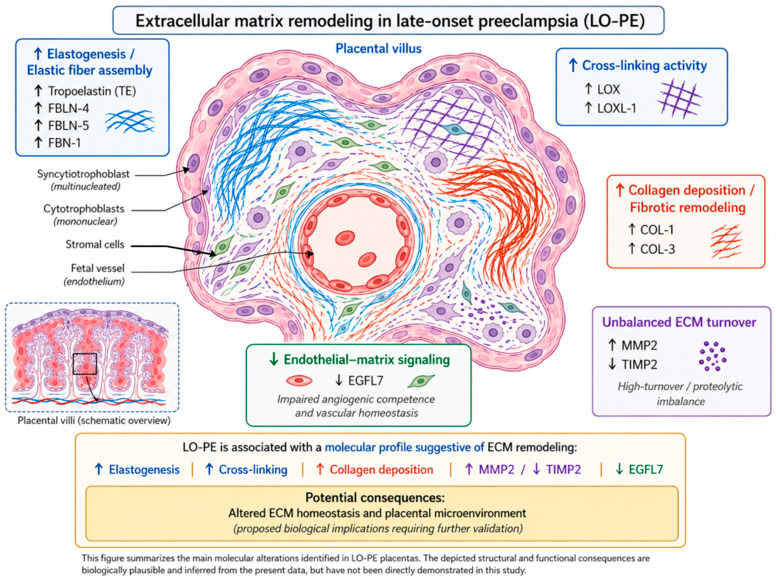
A graphical summary of the results obtained in our study and possible implications in the pathophysiology of late-onset preeclampsia (LO-PE).

From a translational perspective, these findings may have potential diagnostic and therapeutic implications, although they should be interpreted with caution. Since the present study was performed on placental tissue collected at delivery, the identified ECM-related alterations cannot currently be proposed as direct diagnostic biomarkers for clinical use during pregnancy. However, the consistent dysregulation of elastogenesis, collagen deposition, LOX-mediated cross-linking, matrix turnover, and endothelial–matrix signaling suggests that these pathways may contribute to defining specific placental phenotypes in LO-PE (Figure 13). Future studies evaluating whether these ECM-related signatures can be detected in accessible maternal biological samples, such as blood or circulating extracellular vesicles, may help determine their value as candidate biomarkers for risk stratification or disease monitoring. From a therapeutic viewpoint, our results suggest that ECM homeostasis, matrix cross-linking, proteolytic balance, and endothelial–matrix interactions may represent biologically relevant pathways for future mechanistic and preclinical studies. Nevertheless, any diagnostic or therapeutic application would require validation in larger longitudinal cohorts, integration with clinical and Doppler parameters, and functional models able to determine whether ECM remodeling is a driver, consequence, or adaptive response in LO-PE.

### Strengths and Limitations

This study has several strengths. First, it was conducted within a prospective, observational clinical cohort including a well-defined group of women with LO-PE and healthy pregnant controls, classified according to established ACOG criteria. The sample size of 111 placentas provides a solid basis for assessing ECM remodeling in LO-PE. Second, the combination of RT-qPCR and IHC allowed the evaluation of ECM-related alterations at both transcriptional and tissue protein levels, with most markers showing concordant changes between both approaches. Third, placental sampling was performed immediately after delivery and included fragments from different cotyledonary regions, improving tissue representativeness. In addition, immunohistochemical evaluation was independently performed by two blinded histologists, and the statistical analysis included non-parametric testing, correction for multiple comparisons, and correlation analysis to explore ECM network organization.

Some important limitations should also be acknowledged. The observational design prevents the establishment of causal relationships between ECM remodeling and LO-PE pathophysiology. Moreover, placental samples were collected only at delivery, providing information on the final placental phenotype but not on the temporal evolution of these alterations during pregnancy. Although sampling from different cotyledons reduces regional bias, placental heterogeneity may still influence marker expression. In addition, IHC quantification based on the percentage of positive villi provides useful information on marker distribution but does not fully capture staining intensity, subcellular localization or compartment-specific expression. The targeted marker panel allowed focused evaluation of elastogenesis, collagen deposition, cross-linking, matrix turnover and endothelial–matrix signaling but does not provide a comprehensive omics-level characterization of the placental ECM. Furthermore, umbilical artery Doppler parameters were not systematically collected as part of the original study design and therefore could not be incorporated into the present analysis. As Doppler assessment may provide relevant information regarding placental function and fetoplacental hemodynamics, future studies integrating molecular placental findings with Doppler-derived parameters may offer additional mechanistic insight into ECM remodeling in LO-PE. In addition, detailed stratification according to LO-PE severity was not available for all participants, preventing subgroup analyses comparing severe and non-severe LO-PE cases. Future studies should incorporate systematic severity classification to determine whether ECM remodeling patterns differ according to the clinical intensity of the disease. Finally, although gestational age at delivery and cesarean section rate did not differ significantly between groups, maternal age, nulliparity and placental weight showed significant differences and may have influenced the observed ECM profile. These variables may act either as potential confounders or as clinical features intrinsically linked to the LO-PE phenotype, making it difficult to fully disentangle their independent contribution in the present cohort. In particular, the lower placental weight in the LO-PE group may reflect disease-related placental involvement, but it could also contribute to differences in tissue architecture and marker expression. Therefore, future studies integrating larger stratified cohorts, longitudinal designs, adjusted multivariable analyses, digital pathology, compartment-specific approaches and functional models are needed to further clarify the role of ECM remodeling in LO-PE.

## 5. Conclusions

The present findings show that placentas from women with LO-PE exhibit a distinct ECM remodeling profile involving both the elastic and fibrillar compartments of the placental villi. LO-PE placentas displayed increased expression of key elastogenesis-related markers, including TE, FBLN-4, FBLN-5 and FBN-1, together with augmented expression of LOX and LOXL-1, indicating increased activation of elastic fiber assembly and matrix cross-linking pathways. In parallel, the upregulation of COL-I and COL-III reflects increased fibrillar collagen deposition, while the concomitant increase in MMP-2 and reduction in TIMP-2 indicate an imbalance in matrix turnover. The marked decrease in EGFL-7 further supports the presence of altered endothelial–ECM signaling within the placental villous compartment.

In addition, our correlation analysis revealed a bifurcated transcriptional response in LO-PE placentas: whereas the FBLN-4/FBLN-5 axis remained preserved, the broader ECM regulatory network showed a loss of coordinated gene relationships compared with controls, suggesting disruption of the homeostatic integration that normally regulates matrix organization. Overall, these results indicate that LO-PE placentas undergo active structural and molecular ECM remodeling, supporting these mechanisms as a relevant biological feature of LO-PE and a potential contributor to its placental pathophysiology.

## Figures and Tables

**Table 1:** Clinical and demographic characteristics of patients with late-onset preeclampsia and healthy controls. HC: healthy controls; LO-PE: late-onset preeclampsia; n: number of patients evaluated; SD: standard deviation; NS: not significant. Statistical significance was defined as *p* < 0.05 (*) and *p* < 0.0001 (***).

Variable	HC-PW (*n* = 43)	LO-PE (*n* = 68)	*p*-Value
Average age Years ± SD	31.348 ± 5.117	29.015 ± 4.816	* *p* = 0.0154
Nulliparous n (%)	14 (32.56)	53 (77.94)	*** *p* < 0.0001
Average gestational age (weeks ± SD)	39.069 ± 1.486	38.627 ± 1.434	NS
Cesarean section *, n (%)	8 (18.60)	15 (22.06)	NS
Placental weight, Mean ± SD (g)	500.977 ± 65.331	370.254 ± 61.647	*** *p* < 0.0001

* According to the clinical information available to the investigators, none of the cesarean deliveries were performed primarily because of preeclampsia-related complications.

**Table 2:** Primary and secondary antibodies used.

Antigen	Species	Dilution	Provider	Protocol Specifications
TE	Rabbit (Polyclonal)	1:750	Dr Mechan, Washington University School of Medicine, St. Louis, MO, USA	-
FBLN-4	Rabbit (Monoclonal)	1:250	Abcam Cambridge, UK (ab125073)	Citrate tampon in heat (pH = 6.0)
FBLN-5	Rabbit (Polyclonal)	1:1000	Abcam (ab202977)	-
FBN-1	Rabbit (Polyclonal)	1:100	Abcam (ab53076)	Triton 100 × 0.1% in PBS, 10 min
LOX	Rabbit (Polyclonal)	1:500	Dr. Sommer CNRS-UMR (Lyon, France)	Glycine HCl, 30 min RT. 0.2% Hialuronidase, 30 min 42 °C
LOXL-1	Rabbit (Polyclonal)	1:250	Dr. Sommer CNRS-UMR	Glycine HCl, 30 min RT. 0.2% Hialuronidase, 30 min 42 °C
COL-I	Mouse (Monoclonal)	1:400	Sigma-Aldrich St. Louis, MO, USA (C 2456)	-
COL-III	Mouse (Monoclonal)	1:500	Medicorp, Montréal, QC, Canada (AF-5850)	-
MMP-2	Mouse (Monoclonal)	1:1000	NeoMarkers Fremont, CA, USA (CA-4001)	-
TIMP-2	Mouse (Monoclonal)	1:50	Abcam (ab74216)	-
EGFL7	Rabbit (Monoclonal)	1:500	Abcam ab256451	EDTA (pH 9) before incubation with (monoclonal) blocking solution
IgG (secondary)	Mouse (Polyclonal)	1:1000	Sigma-Aldrich (RG-96/B5283)	-

Abbreviations of the molecular markers analyzed: TE: Tropoelastin; FBLN-4: fibulin-4; FBLN-5: fibulin-5; FBN-1: fibrillin-1; LOX: lysyl oxidase; LOXL-1: lysyl oxidase-like 1; COL-I: collagen type I; COL-III: collagen type III; MMP-2: matrix metalloproteinase-2; TIMP-2: tissue inhibitor of metalloproteinases-2. IgG: Immunoglobulin G (secondary antibodies).

**Table 3:** Primers used for RT-qPCR according to each gene: sequences, forward (Fwd) and reverse (Rev), and binding temperatures (T).

GENE	SEQUENCE FWD (5′→3′)	SEQUENCE Rev (5′→3′)	T
TBP	TGC ACA GGA GCC AAG AGT GAA	CAC ATC ACA GCT CCC CAC CA	60 °C
GAPDH	GGA AGG TGA AGG TCG GAG TCA	GTC ATT GAT GGC AAC AAT ATC CAC T	60 °C
ELN (TE)	TTC CCC GCA GTT ACC TTT CC	CTA AGC CAC CAA CTC CTG GG	60 °C
FBLN-4	GTC TTG GAC ATG CCA GGA TTA	TGG AGA TCG TGG GAT AGT TTG	60 °C
FBLN-5	GTC TTG GAC ATG CCA GGA ATA	TGG AGA TCG TGG GAT AGT TTG	58 °C
FBN-1	GGT GAA TGT ACA AAC ACA GTC AGC A	ATA GGA ACA GAG CAC AGC TTG TTG A	60 °C
LOX	GCA GAT GTC AGA GAT TAT GAT CA	ATC GCC TGT GGT AGC CAT AGT	60 °C
LOXL-1	GCA CCT CTC ATA CCC AGG GC	TGG CAG TCG ATG TCC GCA T	60 °C
COL-I	CCA TGT GAA ATT GTC TCC CA	GGG GCA AGA CAG TGA TTG AA	60 °C
COL-III	GAC TTC CAA GAC CTC TTT	CCA CAA GGA TTA CAA GGC TTG	62 °C
MMP-2	ATA ACC TGG ATG CCG TCG TG	CTT CAC GCT CTT CAG ACT TTG G	60 °C
TIMP-2	TCT GGA AAC GAC ATT TAT GG	GTT GGA GGC CTG CTT ATG GG	61 °C

Abbreviations of the molecular markers analyzed: TBP: TATA-binding protein; GAPDH: Gliceraldehyde 3 phosphate dehydrogenase ELN (TE): elastin gene (tropoelastin); FBLN-4: fibulin-4; FBLN-5: fibulin-5; FBN-1: fibrillin-1; LOX: lysyl oxidase; LOXL-1: lysyl oxidase-like 1; COL-I: collagen type I; COL-III: collagen type III; MMP-2: matrix metalloproteinase-2; TIMP-2: tissue inhibitor of metalloproteinases-2.

## Data Availability

The data presented in this study are available on request from the corresponding author. (The data are not publicly available due to privacy or ethical restrictions).

## Supplementary Materials

The following supporting information can be downloaded at: https://www.mdpi.com/article/10.3390/medsci14030364/s1, Table S1: Gene expression results assessed through RT-qPCR; Table S2: Percentage of positive placental villi assessed through immunohistochemistry (IHC).
