# Supplementary material for: Late-Onset Preeclampsia Is Linked to Extensive Remodeling of the Placental Extracellular Matrix
Source: Med Sci (Basel). 2026 Jul 1;14(3):364. doi: 10.3390/medsci14030364 (PMC13413795; doi:10.3390/medsci14030364)
Supplement: Supplementary file 1 [file medsci-14-00364-s001.zip › medsci-4370679-supplementary.pdf]

Supplementary Table S1. Gene expression results assessed through RT-qPCR.

Marker	median_control	IQR_control	median_LOPE	IQR_LOPE	p_value	p_adj	significance
**Tropoelastin**	20,86683	19.606–21.939	29,9382865	25.824–32.686	6,56E-13	8,0178E-13	***
**FBLN_4**	19,038894	18.245–20.584	22,546181	20.855–26.304	6,0026E-12	6,6029E-12	***
**FBLN_5**	20,4589901	19.238–21.54	24,655465	22.634–28.524	9,1632E-12	9,1632E-12	***
**FBN_1**	18,2988989	17.043–19.894	22,482998	21.431–23.582	1,1908E-16	1,8712E-16	***
**LOX**	16,598895	15.822–17.494	22,3289017	20.841–22.493	8,9942E-19	3,299E-18	***
**LOXL_1**	11,38922	10.39–12.479	19,4233679	18.368–21.202	2,7866E-18	7,6632E-18	***
**COL_I**	37,889903	33.446–40.226	45,244902	43.326–51.672	2,4566E-14	3,3778E-14	***
**COL_III**	33,5658986	32.442–34.612	41,323833	40.547–42.507	8,9974E-19	3,299E-18	***
**MMP2**	17,389892	16.373–18.358	21,1896557	20.318–22.415	4,7416E-17	1,0432E-16	***
**TIMP2**	22,148904	20.61–22.502	13,529453	12.027–16.204	8,9942E-19	3,299E-18	***
**EGFL7**	17,2389498	15.086–18.643	10,278841	9.165–11.234	6,1326E-17	1,1243E-16	***

P-values were adjusted for multiple comparisons using the Benjamini–Hochberg false discovery rate.

Supplementary Table S2. Percentage of positive placental villi assessed through immunohistochemistry (IHC)

marker	median_control	IQR_control	median_LOPE	IQR_LOPE	p_value	p_adj	significance
**Tropoelastin**	44	43–47	64	56–67	9,1391E-15	1,0053E-14	***
**FBLN_4**	38	32–43	65	56–68.5	1,2543E-18	2,7596E-18	***
**FBLN_5**	34	29.5–41	65,5	57–71.5	2,7873E-18	4,1759E-18	***
**FBN_1**	33	27.5–34	55	47–57	1,1173E-18	2,7596E-18	***
**LOX**	33	26–34	55	53–58	1,1652E-18	2,7596E-18	***
**LOXL_1**	24	23–25.5	54	47–57.25	8,0517E-19	2,7596E-18	***
**COL_I**	76	67–78	88	85.75–91	2,1971E-11	2,1971E-11	***
**COL_III**	54	46–56	80,5	76–87	8,937E-19	2,7596E-18	***
**MMP2**	24	22–34	54	50–56	4,4647E-18	5,4569E-18	***
**TIMP2**	35	33–39.5	17,5	13–22	2,1777E-18	3,9924E-18	***
**EGFL7**	44	41.5–46	18	13.75–21	3,037E-18	4,1759E-18	***

P-values were adjusted for multiple comparisons using the Benjamini–Hochberg false discovery rate.
